# Investigating pregnant women’s health information needs during pregnancy on internet platforms

**DOI:** 10.3389/fphys.2022.1038048

**Published:** 2022-11-28

**Authors:** Keke Hou, Tingting Hou

**Affiliations:** ^1^ School of Health Sciences, Guangzhou Xinhua University, Guangzhou, China; ^2^ School of Management, Zhengzhou University, Zhengzhou, China

**Keywords:** pregnancy, health information, text analysis, topic analysis, sentiment analysis

## Abstract

Artificial intelligence gives pregnant women another avenue for receiving healthcare information. With the advancement of information and communication technology, searching online for pregnancy information has become commonplace during COVID-19. This study aimed to explore pregnant women’s information-seeking behavior based on data mining and text analysis in China. Posts on maternal and infant-related websites were collected during 1 June 2020, and 31 January 2021. A total of 5,53,117 valid posts were obtained. Based on the data, we performed correlation analysis, topic analysis, and sentiment analysis. The correlation analysis showed the positive effects of population, population with a college education or above, and GDP on post counts. The topic analysis extracted six, nineteen, eighteen, thirteen, eleven, sixteen, thirteen, sixteen, nineteen, and fourteen topics in different months of pregnancy, reflecting different information needs in various pregnancy periods. The results of sentiment analysis show that a peak of the posts emerged in the second month of pregnancy and the proportion of emotionally positive posts reached its peak in the sixth month of pregnancy. The study provides important insights for understanding pregnant women’s information-seeking behavior.

## 1 Introduction

Artificial intelligence (AI) creates opportunities for enabling pregnant women to receive healthcare information. Pregnancy is a crucial period in a woman’s life accompanied by physical change, psychological change, and role transformation. Information-seeking can play an important role in addressing the issue of a healthy delivery. Access to advantageous and concerned information contributes to health-related decisions and the life of both pregnant women and unborn children (Kamali et al., 2018). Childbirth-related information is considerable for performing beneficial interventions and suggestions for pregnant women (Kamali et al., 2018). For example, health-related information will enable women to prepare for pregnancy, concentrate on balanced nutrition and medication use during pregnancy, and make decisions on exercise intensity and mode.

Extant research on health information has addressed the crucial role of research contexts, such as the user group and the domain of information subject in determining information needs (Pian et al., 2020; Reifegerste et al., 2020). The development of information technology and the spread of the mobile Internet enable pregnant women to seek information in a more conveniently and fairly way. Centered on the information needs of maternal health, recent studies have shown that pregnant women’s information-seeking behavior is crucial to enriching the knowledge of childbirth and maternal health and improving maternal health outcomes (Kamali et al., 2018; Ahmadian et al., 2020; Jin et al., 2020; Kassim, 2021). For example, Kamali et al. (2018) found that pregnant women need information such as psychological and physical complications after delivery and pregnancy nutrition in the descriptive study. The qualitative study conducted by Kassim (2021) found that the unavailability of health facilities and limited chances of accessing professional health care could lead to the results that pregnant women seek information from non-professional and informal sources. Ahmadian et al. (2020) identified commonly searched topics during pregnancy using the questionnaire. However, researchers have not treated the topics of information-seeking and pregnant women’s emotions in much detail by employing a relatively large amount of data.

The objective of this research is to explore pregnant women’s information-seeking behavior during the whole pregnancy, including the factors that contribute to the information-seeking behavior, the topics that cause pregnant women’s attention at different months of pregnancy, and the change in pregnant women’s emotions at different stages of pregnancy. By collecting and analyzing the posts in the “pregnant section” under “Mama.cn” from 1 June 2020, to 31 January 2021, and 5,53,117 valid posts, the current work provides a comprehensive study.

## 2 Materials and methods

### 2.1 Data collection

With the advancement of Internet technology, pregnant women’s behavior of seeking online health information has become a universal trend worldwide because of insufficient information received from healthcare providers and the natural advantage of the Internet to ask questions anonymously (Al-Dahshan et al., 2021). As one of the largest maternal and child health websites in China, “Mama.cn” has integrated websites, APPS, new media, micro-network celebrities, and other media resources, covering hundreds of millions of pan-maternal and infant groups. Dedicated to serving all kinds of needs of pregnant women, the company has built several service sections including information, social networking, tools, and e-commerce, aiming to build a diversified Internet maternal, and infant service platform with pregnant women as the core. “Mama.cn” is widely popular among people who are preparing for pregnancy, during pregnancy, and childrearing. In August 2019, “Mama.cn” had 16.479 million active users. The number of active users of “Mama.cn” reached 19.31 million in June 2020, ranking first in the parenting subdivision list in China. Therefore, “Mama.cn” was selected as the research data source for this study. This study collected the posts in the “pregnant section” under “Mama.cn” from 1 June 2020, to 31 January 2021, involving data from “the first month of pregnancy” to “the tenth month of pregnancy.” The current study extracted the following information from the “pregnant section” under “Mama.cn” posts: username, post time, duration of pregnancy, city, and text. A total of 5,75,970 posts were obtained. Examples of our dataset are presented in Table 1.

**TABLE 1:** The examples of dataset.

No	City	Post time	Duration of pregnancy	Text
1	Dazhou city	31/01/2021	Gestation: 3 weeks + 2 days	I just found out I am pregnant, I feel intermittent pain in belly. What's going on?
2	Linyi city	28/01/2021	Gestation: 6 weeks + 1 day	I just went to the toilet and saw a little brown secretion. Not much rubbing, a little worried
3	Wuhan city	25/01/2021	Gestation: 11 weeks + 5 days	My nuchal translucency test passed at one time. The doctor said that the baby was well behaved and in good upgrowth
4	Yinchuan city	31/12/2020	Gestation: 15 weeks + 3 days	My New Year’s resolution is to have a healthy baby! No matter if you are a boy or a girl, stay healthy!
5	Zhongshan city	23/01/2021	Gestation: 29 weeks + 5 days	Sometimes the fetus moves so much. It feels like she is about to jump out from my belly
6	Fuzhou city	27/01/2021	Gestation: 34 weeks + 4 days	34 weeks, I feel pain in public bone, back, and coccyx

We pre-processed the original data before formal analysis by the following procedures. First, the raw information may include missing city tags, irrelevant advertising messages, or posts that did not match the actual time of pregnancy. We filtrated and deleted the above data and finally obtained 5,53,117 texts. Second, the original message may contain distracting information, such as interpunction, emoticons, blank, and hashtags. For excluding data noise and improving data analysis efficiency, we employed regular expressions operations in Python for text filtering.

Measures were performed to ensure data privacy, anonymity, and security. The data collection and analysis did not disclose any privacy issues regarding pregnant women’s identifiable and sensitive information (Favaretto et al., 2020). During data collection, only username, post time, duration of pregnancy, city, and text were extracted. In data processing and analysis, only the duration of pregnancy, city, and text data was used, while the personal information of users was not disclosed. By involving as many samples as possible, more anonymity was preserved as a combination of the variables will be repeated among the samples (Leon-Sanz, 2019).

### 2.2 Methods

#### 2.2.1 Text topic analysis based on latent Dirichlet allocation model

**FIGURE 1:**
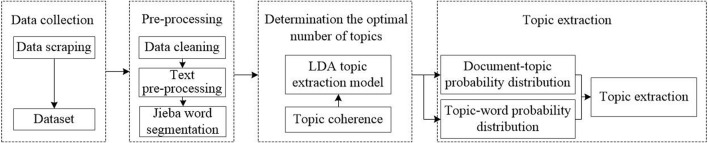
The process of topic extraction.

LDA (Latent Dirichlet Allocation) topic model is a topic probability distribution model based on PLSI (Probabilistic Latent Semantic Indexing) model (Blei et al., 2003). The LDA topic model simulates the process of document generation by using an implied random variable that follows a Dirichlet distribution to represent the document’s topic mixing ratio. Its model structure is more complete and clearer, and the probability inference algorithm is adopted to process the text, which can greatly reduce the dimension of the text representation, to avoid dimension disaster (Blei, 2012). Therefore, LDA is widely used in text mining, text clustering, language processing, and other aspects. The topic number K contained in the document set is a hyperparameter. Given other hyperparameters, the selection process of topic number K is the process of the model searching for the optimal topic number. When the number of topics is too large, there will be many topics without obvious classification semantic information. When the number of topics is too small, broad topics will be generated with a mixture of two or more distributions (Panichella, 2021). Therefore, the determination of the optimal number of topics is an important issue. A coherence score was used to determine the optimal number of topics, with a higher coherence score indicating better quality of topics (Korenčić et al., 2018; Panichella, 2021; Shah et al., 2021). This study used the open-source LDA tool in the Gensim library. The LDA model was evaluated by topic coherence to determine the optimal number of topics. According to the trained LDA model, the topic words under each topic were obtained and the probability of each text belonging to each topic could be directly predicted. Finally, the corresponding topic name was summarized in accordance with the topic words. Figure 1 presents the process of topic extraction in this study.

#### 2.2.2 Text sentiment analysis based on SnowNLP

In recent years, there has been an increasing interest in sentiment analysis (Wang et al., 2019). Sentiment analysis, also known as opinion mining, is an application of text mining and computational linguistics to mine subjective texts with emotional colors and identify the emotional tendencies contained in them. It is a process of identifying information from texts and analyzing, processing, induction, and reasoning subjective texts with emotional color. Through sentiment analysis, researchers can determine users’ emotional orientation in the text. Text-based sentiment analysis methods are mainly divided into three types: sentiment dictionary-based, machine learning-based, and deep learning-based (Xu et al., 2019; Li et al., 2020). The machine learning-based analysis method trains the emotion classifier with emotion-labeled data to achieve emotion classification. Classification accuracy relies on high-quality human-annotated training sets, and large-scale high-quality training data requires a lot of labor costs, and the results of human subjective data annotation will also affect the classification effect. The deep learning-based analysis is based on feature self-learning and deep neural network. It has a good classification effect when dealing with high-dimensional, unlabeled big data, but it is difficult to accurately classify the semantically ambiguous and short text content in social networks. The sentiment dictionary-based method is an unsupervised method, which uses a sentiment dictionary to discriminate the sentiment polarity of text containing keywords, to achieve sentiment classification for each text. There is no need for complex data labeling in the research process and the accuracy of emotion recognition can be improved by adjusting and expanding the vocabulary of the sentiment dictionary according to the specific research background.

SnowNLP, a Python library for Chinese natural language processing, is used to analyze the sentiment of texts. The tool is based on a sentiment dictionary to analyze the sentiment orientation of texts. SnowNLP employs a sentiment dictionary to realize the sentiment tendency analysis of the text. The main functions include part-of-speech tagging, sentiment analysis, keyword extraction, and text summarization (He et al., 2020; Zhang et al., 2021).

## 3 Correlation analysis

The correlation analysis is performed using SPSS24.0. The results of descriptive statistical analysis at the provincial level are presented in Table 2. The data of posts, population, population with a college education or above, illiteracy rate, and GDP of the province are from mainland China. More precisely, population, population with a college education and above, and illiteracy rate are all data from the 2020 census.

**TABLE 2:** The results of descriptive statistical analysis at the provincial level.

Variables	Minimum value	Maximum value	Mean value	Standard deviation	Number (N)
Post counts	410	93449	17819.097	17596.221	31
Population	3648100	126012510	45476733.03	30506939.64	
Population with college education or above (ten thousand)	40	1978	700.6452	443.05752	
Illiteracy rate (%)	0.78	21.11	3.42	3.72	
GDP (100 million yuan)	1902.7	110760.9	32658.5548	26661.80805	

Table 3 presents the correlation analysis results at the provincial level. Post counts was found to positively related to population (ß = 0.889, *p* < 0.001), population with college education or above (ß = 0.835, *p* < 0.001), and GDP (ß = 0.819, *p* < 0.001). However, a significant relationship between post counts and illiteracy rate (*p* > 0.05) was not found in this study. This result is consistent with previous research which indicates that the illiteracy rate had a small and insignificant correlation with computer and Internet penetration rates statistically (Chinn and Fairlie, 2010).

**TABLE 3:** The results of correlation analysis at the provincial level (N = 31).

	Variable	Correlation coefficient	*p*-value
Post counts	Population	0.889***	<0.001
	Population with college education or above	0.835***	<0.001
	Illiteracy rate	−0.227	>0.05
	GDP	0.819***	<0.001

## 4 Topic analysis of information needs

### 4.1 Emerged topics in different months of pregnancy

#### 4.1.1 Information needs in the first month

As mentioned above, topic analysis was divided based on the stages of pregnancy, corresponding to the period from “the first month of pregnancy” to “the tenth month of pregnancy”. Table 4 presents the topics identified in the first month of pregnancy, relative weight, and LDA keywords. Six topics emerged in the first month of pregnancy in which the first frequent topic. “Test strip,” accounts for 20.53% of all topics. “Pregnancy tests consultation,” “early pregnancy inspection,” and “early pregnancy reaction,” accounting for 16.59%, 14.73%, and 14.02%, respectively, were the second, third, and fourth most frequent topics. Among them, early pregnancy reaction refers to pregnant women’s body response during the early pregnancy period. The next two frequent topics are “appeals and desire” and “question for help,” at 12.54% and 10.96%, respectively.

**TABLE 4:** Topics in the first month of pregnancy.

	Topic name	Rate (%)	LDA keywords
1	Test strip	20.53	Last menstrual period, ovulation, pregnancy test paper, deepen, detect, intercourse, color, one deep and one shallow, obvious, ovulatory period
2	Pregnancy tests consultation	16.59	Yes or no, pregnancy, take a look, give a hand, pray, really, two lines, duration
3	Early pregnancy inspection	14.73	Hospital, detect, normal, low progesterone, HCG doubled, draw blood, worry, B ultrasound, brown secretion, blood test
4	Early pregnancy reaction	14.02	Eat, feeling, early pregnancy, collywobbles, everyday, night, emesis, symptom, not good, uncomfortable
5	Appeals and desire	12.54	Baby, hope, mother, good pregnancy, healthy, earnestly hope, love, must, finally
6	Question for help	10.96	Pregnancy, have you ever, discern, circumstance, affect, find, why, need, question

#### 4.1.2 Information needs in the second month

Nineteen topics are identified in the second month of pregnancy. The most frequent ten topics in the second month of pregnancy, relative weight, and LDA keywords are presented in Table 5. The results show that “precautions for early pregnancy,” “early pregnancy inspection,” and “symptoms of early pregnancy” emerged to be the top three frequent topics, accounting for 8.59%, 8.35%, and 7.39%, respectively. The next five frequent topics are “the gender of baby,” “fetal heart and embryo bud,” “vomiting during pregnancy,” “early pregnancy indicators,” and “calculation of pregnancy period,” at 7.04%, 6.54%, 6.40%, 6.31%, and 6.30%, respectively. The following two frequent topics are “appeals and desire” and “prenatal diet,” at 5.70% and 5.34%, respectively.

**TABLE 5:** Topics in the second month of pregnancy.

	Topic name	Rate (%)	LDA keywords
1	Precautions for early pregnancy	8.59	Early stages of pregnancy, purchase, affect, fetus, catch a cold, recommend, create profile, skin care product, attention, nuchal translucency, clothes, prepare
2	Early pregnancy inspection	8.35	Check, B ultrasound, gestational sac, report, show, ectopic pregnancy, *in utero*, yolk, transvaginal ultrasound, germ, recheck
3	Symptoms of early pregnancy	7.39	Feeling, collywobbles, normal, symptom, 6 weeks, 7 weeks, why, once in a while, lower abdominal pain
4	The gender of baby	7.04	Take a look, give a hand, boy, girl, discern, everyone, make out, whether or not
5	Fetal heart and embryo bud	6.54	Fetal heart, embryo bud, hope, healthy, good pregnancy, bless, happy, antenatal care, all the best, *in utero*, rest assured
6	Vomiting during pregnancy	6.40	Vomiting during pregnancy, uncomfortable, reaction, nausea, serious, stomach, anesis, loss of appetite, dizziness, retch
7	Early pregnancy indicators	6.31	Low progesterone, HCG doubled, normal, doctor, draw blood, recheck, decline, blood test, relatively low
8	Calculation of pregnancy period	6.30	Month, day, last menstrual period, count pregnancy period, the last time, intercourse, detect, menstrual cycle, the first day, ovulatory period, pattern
9	Appeals and desire	5.70	Baby, mother, hope, cheer, healthy, love, expectation, grow up, safety, happy, birth
10	Prenatal diet	5.34	Eat, hungry, food, drink, folic acid, loss of appetite, like, not allowed, eat nothing, meat

#### 4.1.3 Information needs in the third month

Eighteen topics are extracted in the third month of pregnancy. Table 6 presents the top ten topics in the third month of pregnancy. The results indicate that “nuchal translucency and filling,” “vomiting during pregnancy,” “the gender of baby,” and “symptom of early pregnancy” emerged to be the four most frequent topics, accounting for 13.05%, 12.51%, 8.65%, and 7.94%, respectively. The next four frequent topics are “prenatal diet,” “fetal heart and embryo bud,” “threatened miscarriage,” and “fetus protection,” at 5.80%, 5.72%, 5.12%, and 4.45%, respectively. The following two most frequent topics are “share and exchange” and “household affairs,” at 4.24% and 4.22%, respectively.

**TABLE 6:** Topics in the third month of pregnancy.

	Topic name	Rate (%)	LDA keywords
1	Nuchal translucency and filling	13.05	Hospital, nuchal translucency, create a profile, need, appointment, prepare, expense, antenatal care, several weeks, empty stomach, draw blood
2	Vomiting during pregnancy	12.51	Vomiting during pregnancy, reaction, serious, food, everyday, stomach, nausea, hungry, loss of appetite, retch, dizziness
3	The gender of baby	8.65	Take a look, girl, boy, everyone, give a hand, curious, make out, discern, checklist
4	Symptom of early pregnancy	7.94	Feeling, collywobbles, normal, bloat, symptom, why, back pain, buttock, lower abdomen, once in a while
5	Prenatal diet	5.80	Eat, drink, prefer, meat, unthink, unable, fruit, spicy, sour, nutrition, appetite, breakfast
6	Fetal heart and embryo bud	5.72	Fetal heart, embryo bud, check, B ultrasound, Last menstrual period, doctor, show, gestational sac, recheck, upgrowth, yolk
7	Threatened miscarriage	5.12	Brown secretion, bleeding, hospital, restroom, find, suddenly, fetus protection, abortion, in hospital
8	Fetus protection	4.45	Doctor, inspection, low progesterone, HCG, progesterone, recheck, take medicine, fetus protection, draw blood, take an injection, suggestion
9	Share and exchange	4.24	Whether or not, expectant mother, expected date of confinement, the same kind, experience, early pregnancy, the same month, exchange, inform, Wechat group
10	Household affairs	4.22	Husband, cry, mother-in-law, work, at home, think, really, not good, marriage, afterwards, mood

#### 4.1.4 Information needs in the fourth month

Thirteen topics are identified in the fourth month of pregnancy. Table 7 presents the top ten topics in the fourth month of pregnancy. “The gender of baby” accounts for 18.98% of all topics. “Down’s syndrome,” “household affairs,” “nuchal translucency,” and “appeals and desire,” accounting for 9.18%, 7.50%, 7.42%, and 7.25%, respectively, were the second, the third, the fourth, and the fifth most frequent topics. The next five frequent topics are “abnormality in antenatal care,” “prenatal diet,” “fetal movement,” “question for help,” and “belly size and weight,” at 6.91%, 6.85%, 6.83%, 6.26%, and 6.25%, respectively.

**TABLE 7:** Topics in the fourth month of pregnancy.

	Topic name	Rate (%)	LDA keywords
1	The gender of baby	18.98	Take a look, boy, girl, everyone, give a hand, curious, nuchal translucency, discern, the first pregnancy, the second pregnancy, want, son, daughter
2	Down’s syndrome	9.18	Inspection, hospital, non-invasive prenatal testing, Down’s syndrome, screening, risks, amniocentesis, suggestions, draw blood, four-dimensional
3	Household affairs	7.50	Husband, child, mother-in-law, mood, work, at home, the first child, home, not good
4	Nuchal translucency	7.42	Nuchal translucency, once, doctor, baby, the first time, successfully, cooperate, finally, twice, make out
5	Appeals and desire	7.25	Baby, hope, mother, healthy, smoothly, cheer, happy, antenatal care, successfully, love, anticipate, bless
6	Abnormality in antenatal care	6.91	Doctor, inspect, worry, B ultrasound, placenta, problem, fetus, bleeding, secreta, upgrowth
7	Prenatal diet	6.85	Eat, food, dislike, drink, hungry, not allowed, unthink, meat, have a meal, specially, loss of appetite
8	Fetal movement	6.83	Feel, belly, move, night, fetal movement, sleep, lie, recently, seem, somewhile, always
9	Question for help	6.26	Pregnant, normal, pain, have you ever, discern, fetal heart, circumstance, why, suddenly, cause, question
10	Belly size and weight	6.25	Pregnant, 3 months, big stomach, almost 4 months, gain, weight, many kilograms, obviously pregnant

#### 4.1.5 Information needs in the fifth month

Eleven topics are extracted in the fifth month of pregnancy. Table 8 indicates the top ten topics in the fifth month of pregnancy. The top two frequent topics are “the gender of baby” and “Down’s syndrome,” at 12.73% and 11.97%. “Fetal movement,” “prenatal diet,” “pregnant women’s physical discomfort,” and “inspection of a large row of deformities” emerged to be the third, fourth, fifth, and sixth frequent topics, accounting for 9.93%, 9.26%, 9.11%, and 8.68%, respectively. The next four most frequent topics are “ponderal growth,” “experience sharing,” “household affairs,” and “appeals and desire,” accounting for 8.09%, 7.34%, 6.95%, and 6.26%, respectively.

**TABLE 8:** Topics in the fifth month of pregnancy.

	Topic name	Rate (%)	LDA keywords
1	The gender of baby	12.73	Take a look, girl, boy, the second pregnancy, give a hand, curious, discern, the first pregnancy, son, daughter
2	Down’s syndrome	11.97	Non-invasive prenatal testing, low risk, Down’s syndrome, smoothly, high risk, DNA, amniocentesis, threshold, suggestion, hope
3	Fetal movement	9.93	Feel, fetal movement, obvious, fetal heart, the first time, seem, normal, once in a while
4	Prenatal diet	9.26	Eat, emesis, pregnancy, food, prefer, everyday, drink, calcium tablet, constipation, meat, hungry, DHA
5	Pregnant women’s physical discomfort	9.11	Night, sleep, legs, buttocks, pain, get up, lie, special, feel ill, uncomfortable, not good, difficulty in sleeping
6	Inspection of a large row of deformities	8.68	Inspection, doctor, hospital, four-dimensional ultrasound, Down’s syndrome, placenta, appointment, antenatal care, Nuchal translucency
7	Ponderal growth	8.09	Pregnant, over 4 months, big belly, weight, gain, kilogram, 5 months, first trimester, without getting fat
8	Experience sharing	7.34	Pregnancy, expected date of confinement, sign in, catch a cold, recommendation, the same kind, exchange, chat, prepare, share
9	Household affairs	6.95	Husband, child, mother-in-law, work, the first child, cry, unthink, in bad mood, look after a baby
10	Appeals and desire	6.26	Baby, mother, hope, cheer, healthy, love, happy, anticipate, bless, birth

#### 4.1.6 Information needs in the sixth month

Sixteen topics are identified in the sixth month of pregnancy. Table 9 shows the top ten topics in the sixth month of pregnancy. The top two topics are “the gender of baby” and “four-dimensional ultrasound,” accounting for 18.65% and 17.38% of all topics. The following four topics, “pregnant women’s physical discomfort,” “prenatal diet,” “household affairs,” and “fetal movement,” comprise 7.53%, 6.33%, 6.03%, and 5.73%, respectively. “Appeals and desire,” “ponderal growth,” “glucose tolerance test,” and “sleep during pregnancy” accounted for 5.55%, 5.21%, 4.79%, and 4.04%, respectively.

**TABLE 9:** Topics in the sixth month of pregnancy.

	Topic name	Rate (%)	LDA keywords
1	The gender of baby	18.65	Take a look, boy, girl, four-dimensional ultrasound results, everyone, give a hand, curious, guess, son, daughter
2	Four-dimensional ultrasound	17.38	Inspection, fetus, four-dimensional ultrasound, worry, problem, normal, umbilical cord, recheck, relatively small
3	Pregnant women’s physical discomfort	7.53	Fetal heart, restroom, bleeding, secreta, pain, feel ill, catch a cold, constipation, serious, afford no relief
4	Prenatal diet	6.33	Eat, prefer, pregnancy, drink, food, calcium tablet, hungry, meat, DHA, breakfast, nutrition, anemia
5	Household affairs	6.03	Husband, mother-in-law, work, at home, cry, unthink, everyday, marriage, boring, insist, work
6	Fetal movement	5.73	Feel, fetal movement, obvious, sometimes, frequent, severe, kick, immovability, belly
7	Appeals and desire	5.55	Baby, mother, hope, girl, boy, healthy, love, cheer, anticipate, birth, bless, all the best, safety
8	Ponderal growth	5.21	Pregnant, over 5 months, kilogram, weight, big belly, fat, gain, control, 6 months
9	Glucose tolerance test	4.79	Hospital, prepare, appointment, glucose tolerance test, expense, drink sugar water, blood glucose, empty stomach, high, normal
10	Sleep during pregnancy	4.04	Night, difficulty in sleeping, everyday, stay awake, uncomfortable, lie, always, tired, sleeplessness, often

#### 4.1.7 Information needs in the seventh month

Thirteen topics are extracted in the seventh month of pregnancy. Table 10 shows the top ten most frequent topics, rates, and LDA keywords. The results present that “the gender of baby” and “pregnant women’s physical discomfort” emerged to be the first and the second most frequent topic, accounting for 21.67% and 13.34% of all topics, respectively. The following four topics are “sleep during pregnancy,” “ponderal growth,” “glucose tolerance test,” and “prenatal diet,” accounting for 7.19%, 6.63%, 6.58%, and 6.49%, respectively. “Items for childbirth,” “household affairs,” “appeals and desire,” and “fetal movement” then comprised 6.36%, 6.09%, 5.82%, and 5.77%, respectively.

**TABLE 10:** Topics in the seventh month of pregnancy.

	Topic name	Rate (%)	LDA keywords
1	The gender of baby	21.67	Four-dimensional ultrasound results, give a hand, take a look, guess, boy, girl, the second pregnancy, the first pregnancy, want, curious
2	Pregnant women’s physical discomfort	13.34	Mid-pregnancy, pain in the legs, pain in the buttocks, uncomfortable, tired, anesis, serious, method, constipation, feel ill
3	Sleep during pregnancy	7.19	Night, sleep, everyday, not good, morning, get up, stay awake, restroom, always, sleeplessness, midnight
4	Ponderal growth	6.63	Big belly, pregnant, kilogram, weight, gain, quick, 6 months, small, fat
5	Glucose tolerance test	6.58	Glucose tolerance, drink sugar water, blood glucose, empty stomach, high, normal, accused of sugar, check, doctor, draw blood
6	Prenatal diet	6.49	Eat, food, prefer, pregnancy, hungry, calcium tablet, fruit, emesis, nutrition, breakfast, meat
7	Items for childbirth	6.36	Expected date of confinement, prepare, purchase, need, goods, maternity package, hospital, clothes, recommend, price, share
8	Household affairs	6.09	Husband, mother-in-law, work, at home, everyday, cry, play with mobile phone, look after a baby
9	Appeals and desire	5.82	Baby, mother, hope, love, healthy, cheer, anticipate, happy, birth, successfully
10	Fetal movement	5.77	Feel, belly, fetal movement, special, normal, recently, obvious, more and more frequent

#### 4.1.8 Information needs in the eighth month

Sixteen topics are identified in the eighth month of pregnancy. Table 11 presents the top ten most frequent topics. As shown in the results, the top two topics are “the gender of baby” and “emotion sharing”, accounting for 10.36% and 10.31%. The following four topics, “prenatal care,” “sleep during late pregnancy,” “prenatal diet,” and “appeals and desire,” account for 7.94%, 7.15%, 6.93%, and 6.76%, respectively. The next four topics are “ponderal growth,” “pregnant women’s physical discomfort,” “household affairs,” and “preparation for delivery,” at 6.47%, 6.43%, 6.26%, and 5.88%, respectively.

**TABLE 11:** Topics in the eighth month of pregnancy.

	Topic name	Rate (%)	LDA keywords
1	The gender of baby	10.36	Take a look, boy, girl, give a hand, the second pregnancy, four-dimensional ultrasound results, curious, daughter, want, the first pregnancy
2	Emotion sharing	10.31	Nervous, anxiety, uncomfortably, smoothly, cheer, emotion, unthink, insist, at home, work, boring
3	Prenatal care	7.94	Inspect, B ultrasound, amniocentesis, too large, too small, position of the fetus, normal, four-dimensional ultrasound, cord around neck, recheck, breech position
4	Sleep during late pregnancy	7.15	Night, sleep, later pregnant trimester, difficulty in sleeping, get up, restroom, stay awake, daytime, wake up in midnight
5	Prenatal diet	6.93	Eat, prefer, drink, hungry, anemia, food, pregnant women, emesis, constipation, meat, nutrition, breakfast, calcium tablet
6	Appeals and desire	6.76	Baby, mother, hope, love, birth, healthy, anticipate, term delivery, father, meet, safety, all the best
7	Ponderal growth	6.47	Expected date of confinement, kilogram, weight, awaiting delivery, control, fat belly, count down, gain
8	Pregnant women’s physical discomfort	6.43	Pain, recently, feel ill, upset stomach, why, lie, later pregnant trimester, tired, sometimes, walk, sit, pain in public bone
9	Household affairs	6.26	Husband, child, mother-in-law, look after the first child, cry, home, marriage, unthink, come back
10	Preparation for delivery	5.88	Buy, hospital, need, clothes, pregnant women, breast pump, goods, recommend, child, prepare, price

#### 4.1.9 Information needs in the ninth month

Nineteen topics are extracted from the ninth month of pregnancy. Table 12 presents the top ten topics, rates, and LDA keywords. The results show that “prenatal care,” “the gender of baby,” and “emotion sharing” emerged to be the top three topics, accounting for 10.78%, 7.96%, and 7.13%, respectively. The next four most frequent topics are “items for childbirth,” “sleep during late pregnancy,” “fetal movement,” and “pregnant women’s physical discomfort” which comprised 6.27%, 6.16%, 6.10%, and 6.05%, respectively. “Prenatal diet,” “household affairs,” and “expected date of confinement” emerged to be the last three topics, at 5.72%, 5.40%, and 4.87%.

**TABLE 12:** Topics in the ninth month of pregnancy.

	Topic name	Rate (%)	LDA keywords
1	Prenatal care	10.78	Doctor, fetal heart, monitor, prenatal care, B ultrasound, fetus, relatively small, normal, biparietal diameter, cord around neck, amniocentesis
2	The gender of baby	7.96	Boy, girl, give a hand, take a look, name, curious, guess, four-dimensional ultrasound results, shape of belly
3	Emotion sharing	7.13	Cheer, anticipate, sign in, count down, insist, the last month, emotion, finally
4	Items for childbirth	6.27	Prepare, purchase, package for delivery, hospital, need, preparation for delivery, goods, clothes, price, delivery, recommendation
5	Sleep during late pregnancy	6.16	Night, sleep, not good, sleeplessness, later pregnant trimester, restroom, daytime, tantalization, last night, midnight, awake
6	Fetal movement	6.10	Belly, fetal movement, recently, whether or not, uterine constraction, terrible, frequent, sometimes, belly firmness
7	Pregnant women’s physical discomfort	6.05	Pain, feel ill, lie, walk, pubis, tired, later pregnant trimester, sit, turn over, get up, buttocks, back pain
8	Prenatal diet	5.72	Eat, pregnant women, prefer, drink, hungry, food, emesis, not allowed, morning, nutrition
9	Household affairs	5.40	Husband, mother-in-law, at home, work, look after, unthink, marriage, child, cook, accompany
10	Expected date of confinement	4.87	Expected date of confinement, pregnant, in advance, over 8 months, day, count, puerperal period, chat

#### 4.1.10 Information needs in the tenth month

Fourteen topics are identified in the tenth month of pregnancy. Table 13 presents the top ten topics in the tenth month. “Appeals and desire” emerged to be the most frequent topics, accounting for 21.17% of all topics. The following five topics, “delivery,” “expected date of confinement,” “pregnant women’s physical discomfort,” “full term,” and “prenatal care,” comprised 13.16%, 7.62%, 6.86%, 6.46%, and 6.19%, respectively. The next four topics are “sleep during late pregnancy,” “nutrition and weight during pregnancy,” “household affairs,” and “good things to recommend,” accounting for 5.96%, 5.64%, 5.28%, and 5.03%, respectively.

**TABLE 13:** Topics in the tenth month of pregnancy.

	Topic name	Rate (%)	LDA keywords
1	Appeals and desire	21.17	Earnestly hope, eutocia, meet, no tear, no side out, safety, throes, super quick, uterine contraction, healthy
2	Delivery	13.16	Uterine contraction, hospital, bleed, stomachache, amniorrhea, the opening of the cervix, boy, girl, have sons and daughters
3	Expected date of confinement	7.62	Expected date of confinement, time, reaction, anxious, no action, steady, delay, 2 days
4	Pregnant women’s physical discomfort	6.86	Belly, pain, feel, fetal movement, pubis, walk, lie, become hard, frequent, waist, sometimes
5	Full term	6.46	Full term, get ready, anticipate, cheer, give birth, count down, finally, nervous, time, insist
6	Prenatal care	6.19	Doctor, inspect, in hospital, amniocentesis, B ultrasound, prenatal care, fetal heart, normal, monitor, worry, fetus, umbilical cord
7	Sleep during late pregnancy	5.96	Night, later pregnant trimester, everyday, difficulty in sleeping, feel ill, tired, tantalization, sleeplessness
8	Nutrition and weight during pregnancy	5.64	Eat, pregnancy, nutrition, weight, grow, pregnant, biparietal diameter, drink, striae gravidarum, control, fat
9	Household affairs	5.28	Husband, child, mother-in-law, look after the first child, at home, expense, work, cry, confinement in childbirth
10	Good things to recommend	5.03	Purchase, compare, recommend, need, choose, paper diaper, clothes, body, prefer, pregnant women, share

### 4.2 Summary of topic analysis about information needs

**FIGURE 2:**
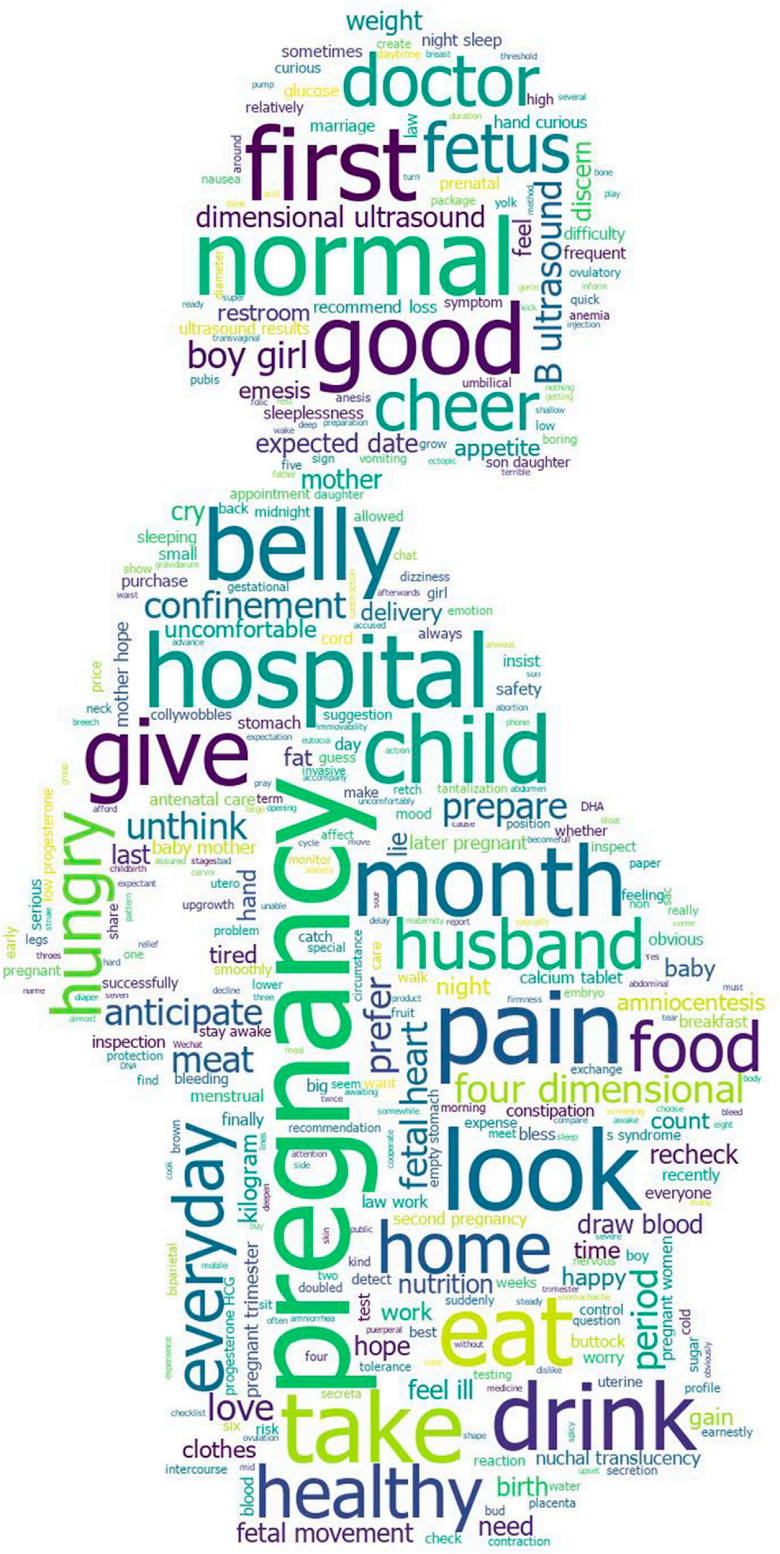
The results of word cloud analysis.

To more vividly show the main topics that pregnant women pay attention to during the whole pregnancy, we conducted a word cloud analysis on the LDA keywords of the topics during pregnancy. The results are presented in Figure 2. In word cloud statistics, word frequency is distributed by font size. As shown in Figure 2, the fonts of words such as “pregnancy,” “child,” and “fetus” are prominent, indicating that the topic of pregnancy is centered on pregnant women and babies. Secondly, the fonts of words such as “hospital,” “normal,” “doctor,” and “healthy” are also clearly displayed, indicating that obstetric examination is an important topic that pregnant women continue to pay attention to during pregnancy, which can help pregnant women to keep abreast of their physical status and fetal upgrowth. Then, words such as “pain,” “belly,” “good,” “eat,” “hungry,” “drink,” and “food” appeared frequently, reflecting pregnant women’s concerns about their physical condition and diet during pregnancy. Words such as “cheer,” “hope,” “happy,” “love,” “boy,” and “girl” reflect pregnant women’s good wishes for their babies and their curiosity about their babies’ gender.

## 5 Sentiment analysis

**FIGURE 3:**
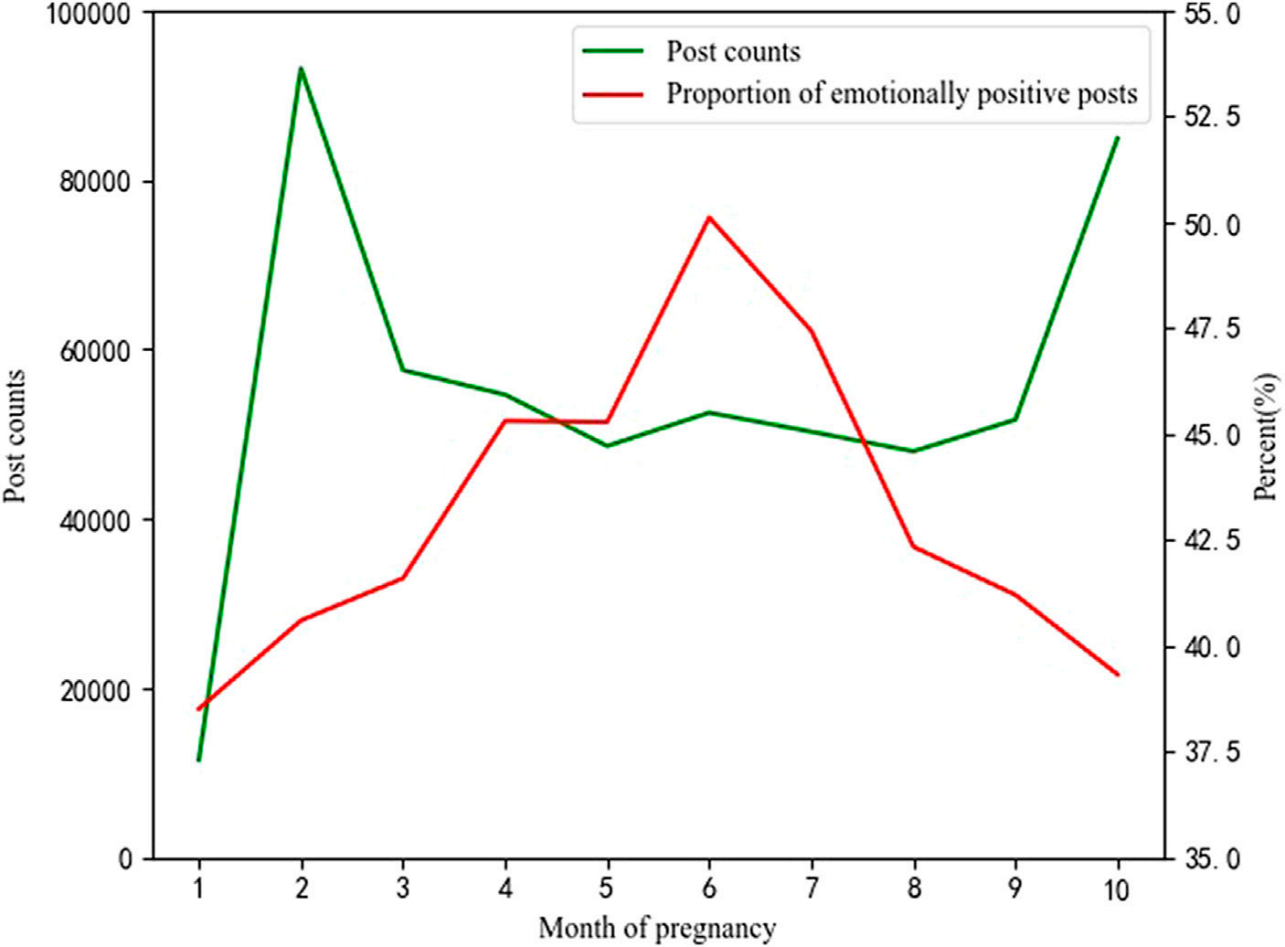
The results of sentiment analysis.

Sentiment analysis is performed to further understand the changes in pregnant women’s information-seeking behavior during pregnancy. As discussed earlier, we use Python to call the third-party library SnowNLP to calculate the sentiment value of each post text, and the range of sentiment value results is [0, 1]. Among them, a sentiment with a value greater than 0.5 is positive, and a sentiment less than or equal to 0.5 is negative. The closer the value is to 1, the more positive the emotion; the closer the value is to 0, the more negative the emotion. Figure 3 presents the posts with a sentiment value greater than 0.5 in each pregnancy month.

By combining the outcomes of topic analysis and sentiment analysis, the results show that in the first month of pregnancy, the number of posts is relatively small, mainly focusing on topics such as “test strip” and “pregnancy tests consultation,” and the proportion of emotionally positive posts is also relatively low. On the one hand, many pregnant women have not found out that they are pregnant in the first month of pregnancy; on the other hand, the first month of pregnancy is often unstable and at a loss for pregnant women, so their emotions are relatively negative.

The number of posts in the second month of pregnancy is the most, but the proportion of posts with positive emotions is also relatively low. In the second month of pregnancy, most pregnant women have already guessed or confirmed pregnancy, but new pregnant women have little knowledge about pregnancy. Therefore, posts about “precautions for early pregnancy,” “early pregnancy inspection,” “symptoms of early pregnancy” and other related early pregnancy topics surged. However, due to the uncertainty of the baby’s status and the lack of relevant knowledge of pregnant women, the proportion of emotionally positive posts in the second month of pregnancy is relatively low. After the first 2 months of relevant inspections and understanding of pregnancy knowledge, pregnant women have entered a relatively mature stage. At the same time, the status of the baby gradually stabilized, so the number of posts from the second month of pregnancy to the third month dropped significantly, and it continued to be stable until the ninth of pregnancy.

The proportion of emotionally positive posts from the third month to the ninth month of pregnancy is higher than that in other months, and there is an upward trend from the third month to the sixth month of pregnancy. The proportion is the highest in the sixth month of pregnancy, and then gradually decreases. After the third month of pregnancy, the baby’s state gradually stabilizes, the pregnant women’s belly gradually bulges, and the pregnant women can even feel the baby’s fetal movement, but there is generally no obvious physical discomfort, so the pregnant women’s emotions are relatively more positive. Since the seventh month of pregnancy, the baby’s weight increases, the pregnant women’s belly increases, the body gradually becomes clumsy, and the body also has various discomforts such as soreness and difficulty sleeping, so pregnant women show more negative emotions.

The number of posts in the tenth month of pregnancy surged again, second only to the second month of pregnancy, and the proportion of emotionally positive posts also dropped sharply, only higher than in the first of pregnancy. The tenth month of pregnancy is the month when the baby is about to be born. On the one hand, the pregnant women’s body aches and sleep problems are more prominent. On the other hand, pregnant women are faced with the uncertainty of childbirth, and a state of fear and anxiety appears. It can also be seen from the results of the topic analysis that in the current month, “appeals and desire” ranked first among the topics that pregnant women paid attention to, accounting for 21.17%. In addition, “expected date of confinement” and “pregnant women’s physical discomfort” are also the main contents of concern for pregnant women.

## 6 Discussion and conclusion

### 6.1 Summary of findings

The purpose of the current study was to investigate pregnant women’s information-seeking behavior. By a combination of descriptive analysis, topic analysis, and sentiment analysis, the current work expands our knowledge by proving important findings. The correlation analysis showed that more pregnant women contribute to more posts. Moreover, pregnant women with a college education or above are more likely to seek information about pregnancy on internet platforms. The more economically developed cities have higher Internet usage. Therefore, pregnant women will be more probable to use Internet platforms to seek information.

Furthermore, the topics from the first month to the tenth month of pregnancy were extracted in topic analysis. The findings show that the topics in different months of pregnancy relate to the present stages of pregnancy. The current paper identified six, nineteen, eighteen, thirteen, eleven, sixteen, thirteen, sixteen, nineteen, and fourteen topics in different months of pregnancy. The specific topics in different stages show the changes in pregnant women’s attention.

In addition, the sentiment analysis showed the variation of pregnant women’s emotions in information-seeking. The results of sentiment analysis show a peak of the posts in the second month of pregnancy. The proportion of emotionally positive posts reached its peak in the sixth month of pregnancy. Pregnant women’s emotional sentiment deeply interacts with the results of topic analysis.

### 6.2 Practical and theoretical implications

Our study presents theoretical and practical significance. First, this is one of the first studies to understand pregnant women’s information-seeking using the methods of data mining and text analysis. Previous studies on the information needs of maternal health revealed the topics that pregnant women pay attention to; however, the existing work is limited in the descriptive analysis and self-reported questionnaire data (Kamali et al., 2018; Ahmadian et al., 2020; Jin et al., 2020; Kassim, 2021). This study is unique by employing enormous quantities of data and the research data covers a long period. By visualizing the posts of every province, the geographical distribution of pregnant women’s posts was clearly displayed. The current study enriches our understanding of the relationships among pregnant women’s information-seeking, regional economic development level, and educational level.

Second, this study provides comprehensive research, involving abundant analysis. Compared with previous research (Kamali et al., 2018), the current work divides the data from the first month of pregnancy to the tenth month of pregnancy and analyzes the large amounts of data according to the pregnancy period. This study provides important insights for understanding the change of emotions during different pregnant stages and connecting the changes of emotions with the topics that cause pregnant women’s attention. The current work provides the perspectives for future research by the subdivision of data in different pregnant stages.

Third, the findings of this study have several practical implications. The findings indicate that pregnant women pay attention to different topics during various months of pregnancy. The maternal and infant-related websites should provide customized information recommendations for pregnant women according to their stages of pregnancy. For example, information such as precautions and inspection for early pregnancy should be recommended for pregnant women in the second month of pregnancy. Moreover, the proportion of emotionally positive posts reached its peak in the sixth month of pregnancy and is relatively low in the first and the tenth of pregnancy. The relevant government management departments and hospitals should concern about anxiety during early pregnancy and before delivery. The popularization of knowledge about pregnancy and childbirth would be useful for improving pregnant women’s emotions.

### 6.3 Limitations and future research

The study is subject to several inevitable limitations. First, the data source of this study is “Mama.cn” mainly located in China. What is now needed in the future is a cross-national study involving data for countries at different levels of development. The present study lays the groundwork for future research into pregnant women’s information-seeking behavior around the world. Future studies are encouraged to improve the generalizability of the current work by involving data from different countries and understanding the role of cultural identity in determining pregnant women’s information-seeking. Second, the data such as personal attributes and specific family environments are not included in the paper since such data cannot be obtained from the website. It would be interesting to investigate the effect of family-related variables on pregnant women’s emotional sentiment in future work.

## Data Availability

The original contributions presented in the study are included in the article/supplementary material, further inquiries can be directed to the corresponding author.
